# Editorial: Nursing perspectives in mental health and psychiatric disorders in all patients

**DOI:** 10.3389/fpsyg.2023.1264438

**Published:** 2023-11-20

**Authors:** Elsa Vitale, Selmin Köse, Yun-Chen Chang

**Affiliations:** ^1^Local Heath Authority (ASL) of Bari, Bari, Italy; ^2^Nursing Department, Faculty of Health Science, Biruni University, Istanbul, Türkiye; ^3^School of Nursing and Graduate Institute of Nursing, China Medical University, Taichung City, Taiwan; ^4^Nursing Department, China Medical University Hospital, Taichung City, Taiwan

**Keywords:** nursing, perspectives, psychiatric disorders, patients, mental health

There is no universally agreed-upon definition of mental health. Generally, an individual's lifestyle or habit could include signs to his or her mental health. Often, mental health was considered as an emotional, psychological, and social wellness condition, characterized by sufficient perception in interpersonal relationships, effective behavior and coping, positive self-concept, and emotional stability (Galderisi et al., 2015). Mental health could be influenced by individual, interpersonal, and social/cultural factors. Specifically, individual, or personal, factors regarding an individual's biologic composition, autonomy, self-esteem, capacity for growth, vitality, ability to find meaning in life, emotional resilience or hardiness, sense of belonging, reality orientation, and coping or stress management skills (Bhugra et al., 2021). Interpersonal or relationship, elements could involve effective communication, capability to help others, intimacy, and a balance of separateness. Finally, social/cultural, and environmental, factors could include a sense of community, access to adequate resources, intolerance of violence, support of diversity among people.

Promotion and prevention interventions activity for both individuals and groups, by recognizing the individual, social and structural determinants of mental health, and then, inducing to decrease risks, could improve cope strategies and establish comfortable work environment (Compton and Shim, 2020).

The present Research Topic aimed to explore nursing perspectives within mental health psychiatric disorders in order to promote, protect and restore mental health through all areas of nursing, in all ages of the patient by a complex interplay of individual, social and structural stresses and vulnerabilities. In this scenario it was emphasized the importance of different social supports among young and middle-aged kidney transplant recipients (Hu et al.) or among patients with multiple sclerosis (MS) (Kołtuniuk et al.). Marital status, education level, residence, *per capita* monthly income, medical insurance, work status, post-transplant time, body mass index, creatinine status, and complications were all associated to psychosocial adaptation (*p* < 0.05). While, a negative illness perception appeared to negatively impacted on their social support (Kołtuniuk et al.).

As regards social support, parents represented an important source in the psychosocial care of their children (Mackova et al.). However, previous studies showed the parents' roles in care without considering the perspective of care providers. Four main themes were described belonging to parents' caring positions, such as: parents as a source of adolescents' troubles; parents trying to avoid from duties for their children; parents as an engaged caring component for their sick children; and parents as an obstacle to effective necessary care. Therefore, more selected interventions through multi-problem family approaches were needed to support parents in their children care. By considering patients' positions, higher incidences in psychiatric disorders, such as mood and depression were also associated to the quality of life (QOL) perceptions. For example, among patients suffering from multiple sclerosis (MS), findings suggested that more than half of the respondents (68%) suffered from depression at several levels. The perception of QOL was influenced by the number of complaints, daily living activities, depressive symptoms, higher education, and material status (*p* < 0.005) (Kołtuniuk et al.). Beyond all the psychiatric disorders abovementioned, an emergent psychiatric disorder, namely the Fear of birth (FOB) was becoming increasingly which impacted on both the maternal and infant health by also influencing women's fertility desires (Zeng et al.). Three themes in the participants' experience with fear of birth were highlighted: an invisible dilemma in fear of all sides, the choice to give birth naturally, live with awaken of maternal spirit by hoping in bloom, Chinese tolerance culture, and obstetric analgesia. Fear of birth was recognized as a complex emotion, associated to loneliness, ambivalence and fear emotions.

Finally, we could not forget the COVID-19 pandemic which negatively influenced mental health and quality of life (QoL) among people all around the world (Vitale et al., 2022). In this regard, among 618 Macau residents, mental health disorders and QoL were explored, particularly the interconnection between different depressive symptoms and QoL among Macau residents during this period (Si et al.). Data showed that participants with depression reported lower QoL (*p* = 0.019). Therefore, interventions in cognitive behavioral therapy should be improved in depressive participants.

In conclusion, promotion and prevention interventional activities for both individuals and groups, by recognizing the individual, social and structural determinants of mental health, and then inducing to decrease risks, could improve cope strategies and establish comfortable cating environments for mental health both for patients and their social supporters. On the other hand, promotion and prevention schedules advocating and initiating by healthcare workers, should include the education, training, environment, housing, and welfare fields.

## Author contributions

EV: Conceptualization, Writing—original draft, Writing—review & editing. SK: Supervision, Writing—review & editing. Y-CC: Supervision, Writing—review & editing.
